# Nanocrystalline/Amorphous Tuning of Al–Fe–Nb (Mn) Alloy Powders Produced via High-Energy Ball Milling

**DOI:** 10.3390/ma17225627

**Published:** 2024-11-18

**Authors:** Nguyen Thi Hoang Oanh, Dao Truong An, Nguyen Hoang Viet

**Affiliations:** School of Materials Science and Engineering, Hanoi University of Science and Technology, Hanoi 100000, Vietnam; oanh.nguyenthihoang@hust.edu.vn (N.T.H.O.); daotruongan.smse@gmail.com (D.T.A.)

**Keywords:** amorphous alloys, nanocrystalline alloys, mechanical alloying, magnetic property, thermal stability, Al–TM alloys, phase transformation

## Abstract

The demand for advanced Al-based alloys with tailored structural and magnetic properties has intensified for applications requiring a high thermal stability and performance under challenging conditions. This study investigated the phase evolution, magnetic properties, thermal stability, and microstructural changes in the Al-based alloys Al_82_Fe_16_Nb_2_ and Al_82_Fe_14_Nb_2_Mn_2_, synthesized via mechanical alloying (MA), using stearic acid as a process control agent. The X-ray diffraction results indicated that Al_82_Fe_16_Nb_2_ achieved a β-phase solid solution with 13–14 nm crystallite sizes after 5 h of milling, reaching an amorphous state after 10 h. In contrast, Al_82_Fe_14_Nb_2_Mn_2_ formed a partially amorphous structure within 10 h, with enhanced stability with additional milling. Magnetic measurements indicated that both alloys possessed soft magnetic behavior under shorter milling times (1–5 h) and transitioned to hard magnetic behavior as amorphization progressed. This phenomenon was associated with a decrease in saturation magnetization (M_s_) and an increase in coercivity (H_c_) due to structural disorder and residual stresses. Thermal stability analyses on 10 h milled samples conducted via differential scanning calorimetry showed exothermic peaks between 300 and 800 °C, corresponding to phase transformations upon heating. Post-annealing analyses at 550 °C demonstrated the presence of phases including Al, β-phase solid solutions, Al₁_3_Fe₄, and residual amorphous regions. At 600 °C, the Al_3_Nb phase emerged as the β-phase, and the amorphous content decreased, while annealing at 700 °C fully decomposed the amorphous phases into stable crystalline forms. Microstructural analyses demonstrated a consistent reduction in and homogenization of particle sizes, with particles decreasing to 1–3 μm in diameter after 10 h. Altogether, these findings highlight MA’s effectiveness in tuning the microstructure and magnetic properties of Al–Fe–Nb (Mn) alloys, making these materials suitable for applications requiring a high thermal stability and tailored magnetic responses.

## 1. Introduction

In recent decades, the rapid development of advanced materials has highlighted the need for novel structural and functional materials, which has become a core area of study in materials science [1,2]. Aluminum–transition metal (Al–TM) alloys have become increasingly significant in this field due to their outstanding properties, such as a high strength-to-weight ratio, superior corrosion resistance, and impressive thermal stability at elevated temperatures in challenging environments, including oxidizing, carburizing, and sulfidizing conditions [3,4]. These advantageous qualities make Al–TM alloys ideal for applications across diverse manufacturing sectors, including the automotive, aerospace, and electronics industries. Among these, Al–Fe–Nb and Al–Fe–Nb (Mn) alloys are particularly compelling to researchers, not only for their corrosion resistance in harsh environments but also for their enhanced mechanical strength. This improvement is achieved by tailoring the nanostructures to include specific combinations of nanocomposites, and amorphous and nanocrystalline phases. Nevertheless, a significant challenge remains in designing and controlling these nanostructures to optimize their properties, which currently limits broader technological applications. Advancing methods for nanocrystalline/amorphous tuning will be essential to fully realize the potential of these alloys in industrial settings [2,4,5,6,7,8].

Within these Al-based systems, the addition of niobium (Nb) plays a significant role in enhancing the microstructure and stability of the alloy. While Nb and Al share similar atomic radii (both are approximately 143 pm), Nb contributes to alloy stability not via size-induced lattice strain but through its high melting point, chemical stability, and limited solubility in aluminum [9,10]. The presence of Nb often promotes the formation of finely dispersed second-phase particles within the aluminum matrix. These particles then act as obstacles to grain boundary movement and dislocation motion, thereby improving mechanical strength and thermal stability [7,11,12,13]. Moreover, Nb encourages the formation of stable intermetallic compounds such as Al_3_Nb, which are known to enhance high-temperature performance and oxidation resistance [13,14,15]. These properties make Nb an ideal alloying element for applications requiring prolonged thermal stability.

One of the most effective methods for fabricating these materials is the mechanical alloying (MA) method. MA is an advanced material handling technique that combines mechanical and chemical processes to create materials with unique structures and compositions. In the MA process, heavy plastic deformation, fracturing, and the cold welding of powders enhance atomic diffusion and induce the formation of the nanocrystalline/amorphous phase [16,17,18,19,20,21,22]. The accumulation of deformation energy and lattice defects increases the free energy of the crystalline phases and thus destabilizes them relative to the amorphous state. This process usually occurs in systems with large negative mixing enthalpy, as is the case with many Al–TM alloys. According to research by Suryanarayana et al. [18], the Al–Zr system has a negative mixed enthalpy of -44 kJ/mol, which facilitates the formation of amorphous structures. Notably, MA can produce an amorphous structure under a lower concentration of alloy elements than traditional rapid-casting methods, expanding the range of components that can achieve an amorphous structure.

In this study, we explore the structural evolution, phase transitions, magnetic characteristics, and thermal stability of nanocrystalline and amorphous Al_82_Fe_16_Nb_2_ and Al_82_Fe_14_Nb_2_Mn_2_ alloys produced via mechanical alloying. Detailed analyses of these alloys, including their microstructural transformations and property enhancements, provide insights into the mechanisms underlying phase stability and alloy performance in demanding applications.

## 2. Materials and Methods

**Figure 1:**
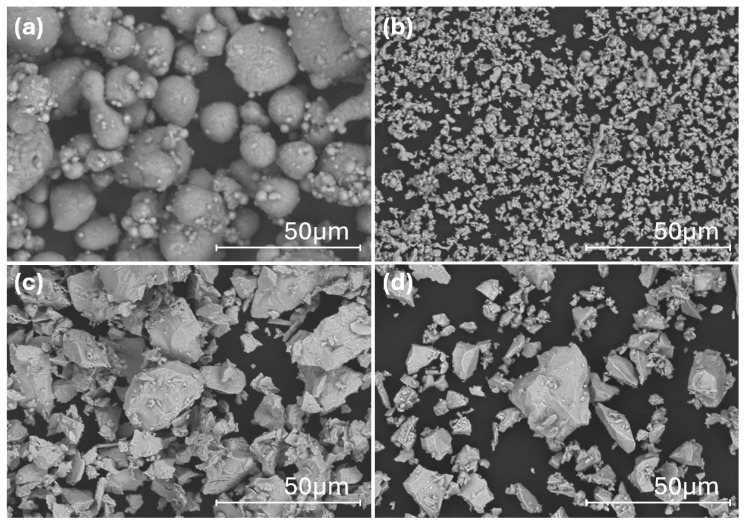
Surface morphology of starting elemental powders of (**a**) Al, (**b**) Fe, (**c**) Nb, and (**d**) Mn.

Elemental powders of aluminum (Al), iron (Fe), niobium (Nb), and manganese (Mn) (Sigma-Aldrich, Dorset, UK), each with a purity of 99.9% and an average particle size of less than 45 μm, were used for MA alloy synthesis. The surface morphology of the elemental powders is shown in Figure 1. The target compositions for this study were Al_84_Fe_16_Nb_2_ and Al_82_Fe_14_Nb_2_Mn_2_. Mixing and alloying were carried out in a high-energy planetary ball mill (AGO-II type) utilizing sealed hardened steel vials and tungsten carbide (WC) balls to prevent contamination. The milling process was conducted in an argon atmosphere to minimize oxidation, using a milling rotation speed of 350 rpm with a ball-to-powder weight ratio of 20:1 and milling times of 1, 2, 5, and 10 h. To prevent powder adherence to the milling tools (ball, powder, and vial wall), 3 wt.% stearic acid was added as a process control agent.

The thermal behavior of the resulting powders was analyzed using a Setaram Labsys Evo S60/58988 Differential Thermal Analyzer via differential scanning calorimetry (DSC) at a heating rate of 20 K/min, allowing us to observe changes in thermal properties with temperature. Based on the DSC results, the as-milled powders were annealed in quartz tubes under a controlled argon atmosphere to maintain an inert environment and prevent oxidation. Annealing was performed at temperatures of 550 °C, 600 °C, and 700 °C, with an argon gas flow rate of 70 mL/min. Heating was controlled at a rate of 10 K/min up to the target temperatures, with the samples held for durations of 15 min to achieve uniform heat treatment. After annealing, the furnace was allowed to cool naturally under continuous argon flow.

The phase composition of the powders was characterized using an X’Pert PRO diffractometer with Cu Kα radiation and scanned over an angular range of 20° ≤ 2θ ≤ 100°. The phases present in the alloy samples were identified using the MDI Jade 6.5 software, providing detailed phase analysis and insight into the structural evolution of the alloy compositions following MA and heat treatment.

The morphology and elemental composition of the as-milled and annealed powders were examined using a scanning electron microscope (SEM) equipped with energy-dispersive X-ray spectroscopy (EDS). SEM/EDS analyses were conducted at various magnifications to observe the particle shapes and morphology in the alloy powders. EDS was used for elemental analysis to identify the distribution and relative concentrations of alloying elements within individual particles.

The magnetic properties of the samples were evaluated using a vibrating sample magnetometer (VSM). Measurements were performed at room temperature, 298 K, with a magnetic field strength of up to ±10 kOe to assess magnetic characteristics such as coercivity (H_c_), saturation magnetization (M_s_), and remanence (M_r_). These magnetic measurements provided insights into the structural and compositional effects on the magnetic properties of the nanocrystalline and amorphous phases induced by mechanical alloying and subsequent annealing.

## 3. Results and Discussion

**Figure 2:**
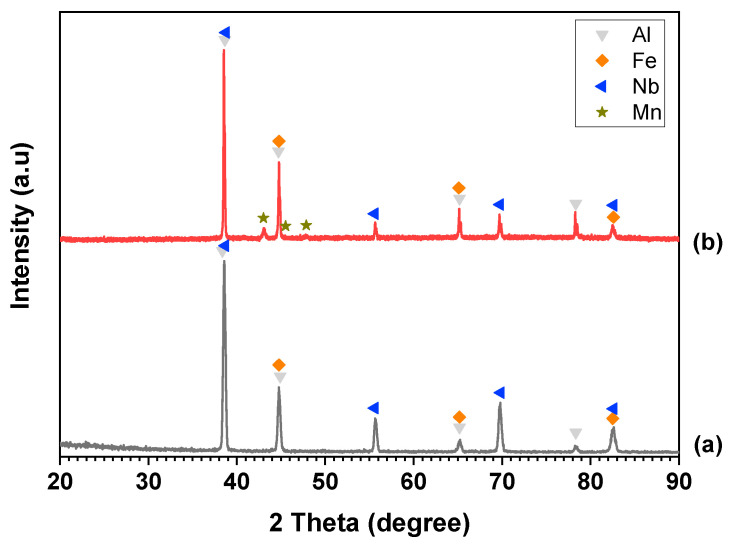
XRD patterns of starting mixture powders of (**a**) Al–Fe–Nb and (**b**) Al–Fe–Nb–Mn.

**Figure 3:**
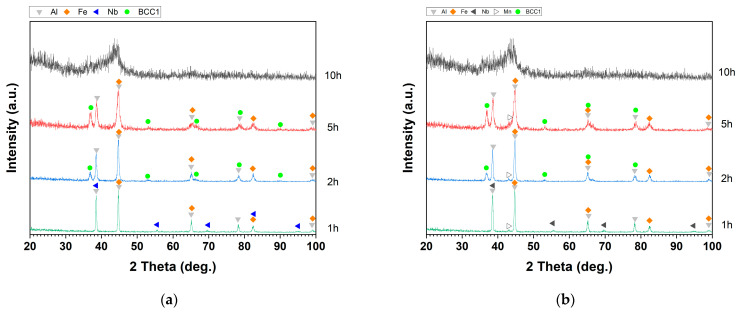
XRD patterns of (**a**) Al_82_Fe_16_Nb_2_ and (**b**) Al_82_Fe_14_Nb_2_Mn_2_ powders milled for different milling times.

Figure 2 presents the XRD patterns of the Al–Fe–Nb and Al–Fe–Nb–Mn mixed powders before milling, where the diffraction peaks represent pure elemental Al, Fe, Nb, and Mn. The diffraction angles (2 theta) and intensities matched the standard reference cards of Al (PDF #04-0787), Fe (PDF #06-0696), Mn (PDF #33-0887), and Nb (PDF #34-0370). The XRD patterns of Al_82_Fe_16_Nb_2_ and Al_82_Fe_14_Nb_2_Mn_2_ alloy powders milled at different times are presented in Figure 3 (1, 2, 5, and 10 h). After 1 h of milling, the diffraction peaks still indicated the presence of pure metals in both alloys. With an increase in the milling time to 2 h, the intensity of the diffraction peaks of pure elements decreased, and the width of the peak became broader. At this time, the crystalline size reduced because the fracture process dominated. However, the diffraction peaks of Nb disappeared, and new diffraction peaks of the solid solution phase β_ss_ became observable. After 5 h of milling, the diffraction peaks of the elements expanded significantly. Moreover, the intensity of the BCC solid solution (BCC1) phase increased compared to the intensity levels of the 2 h as-milled samples. Notably, the characteristic peaks of Al, Fe, Nb, and BCC1 were no longer visible in the XRD patterns of the Al_82_Fe_16_Nb_2_ alloy. Only a halo peak characterized by an amorphous phase was visible. The amorphous phase formation in the Al_82_Fe_14_Nb_2_Mn_2_ alloy was similar to that of the Al_82_Fe_16_Nb_2_ alloy. However, after 10 h of milling, the Al_82_Fe_14_Nb_2_Mn_2_ alloy reached a partial amorphous phase state. Replacing 2 at.% of Fe atoms with Mn in the Al_82_Fe_16_Nb_2_ alloy affected the formation of the amorphous phase. The amorphization process of the Al_82_Fe_16_Nb_2_ alloy system was completed after 10 h milling due to the relatively high negative mixing enthalpy of the Al–Fe, Al–Nb, and Fe–Nb pairs of −11, −18, and −11 kJ/mol, respectively, as shown in Table 1.

The Al_82_Fe_16_Nb_2_ alloy only satisfies rules (1) and (2) of Inoue’s three empirical rules for the formation of bulk amorphous materials [23,24,25]. Since the atomic size of Nb is equal to that of Al, the size difference of Nb–Al is 0%. According to research by Oanh et al. on the Al–Fe–Ni(Ti, Cu) material system, negative mixing enthalpy is the most essential factor among the three empirical rules when preparing amorphous alloys using MA [26]. For the Al_82_Fe_14_Nb_2_Mn_2_ alloy system, all the element pairs had negative mixing enthalpy, except for the Fe–Mn pair, which had a mixing enthalpy of 0 kJ/mol. This result explains why substituting 2 at.% Fe with Mn resulted in an alloy that was not completely amorphous after 10 h of milling. The crystalline size of BCC1 phase for Al_82_Fe_16_Nb_2_ and Al_82_Fe_14_Nb_2_Mn_2_ alloys after milling for 2 and 5 h was calculated using the Scherrer formula [27], as outlined in Table 2. The crystalline size of the solid solution BCC1 phase was about 13–15 nm.

**Table 1:** Atomic radii mismatch (in %) and enthalpies of mixing (in kJ/mol) for binary systems involving Al, Fe, Nb, and Mn [25,28].

	Al	Fe	Nb	Mn
Al	-	13.2 [%]	0 [%]	21.6 [%]
Fe	−11 [kJ/mol]	-	13.2 [%]	9.6 [%]
Nb	−18 [kJ/mol]	−16 [kJ/mol]	-	21.6 [%]
Mn	−19 [kJ/mol]	0 [kJ/mol]	−4 [kJ/mol]	-

**Figure 4:**
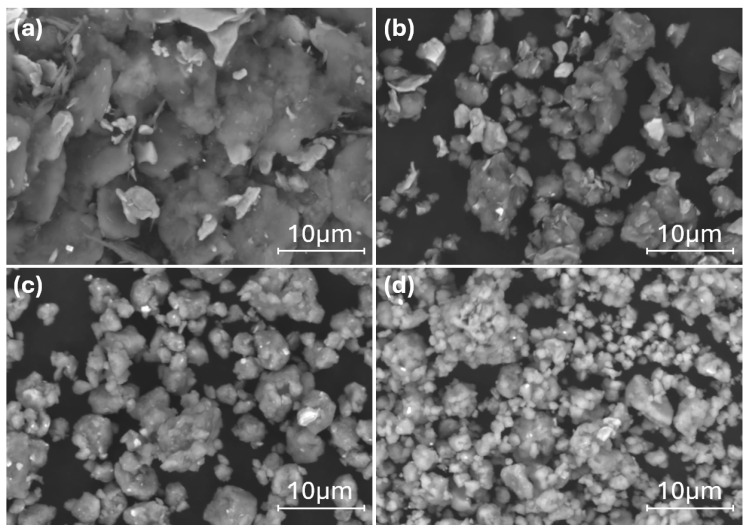
SEM micrographs of the Al_82_Fe_16_Nb_2_ powders milled for (**a**) 1 h, (**b**) 2 h, (**c**) 5 h, and (**d**) 10 h.

SEM images of Al_82_Fe_16_Nb_2_ powders after different milling times are presented in Figure 4. In the early stage of the milling process (1 h), the mixture of Al, Fe, and Nb elemental powders was deformed due to collision with the milling balls and milling vial. The plasticity of Al, Fe, and Nb flattened these elements after impact. At this stage, the cold-welding process dominated the adhesion metal powders comprising the layer structure (Figure 4a). The white phase corresponds to the Nb metal inlaid and adhering to the dark phase (Al and Fe metals). The size of the as-milled powder was about 50 μm. With an increase in the milling time to 2h, the milling powder experienced a fragmentation process, reducing the particle size to less than 10 μm (Figure 4b). This decrease in particle size was evident with a decrease in crystal size due to the diffraction peak being broadened compared to that of the sample after 1 h of milling (Figure 3a). With an increase in the milling time to 5 h, the fragmentation process dominated the powder size by about 5 μm (Figure 4c). The powder particles then became more uniform compared to the results after 2 h of milling. According to the XRD patterns in Figure 3a, a new phase of the solid solution β-phase (BCC1) began to form alongside a reduction in crystal size (due to the diffraction peak being broader than that of the 2 h as-milled sample). Thus, increasing the milling time to 5 h promoted diffusion, leading to the formation of a solid solution of BCC1. With an increase in the milling time to 10 h (Figure 4d), the powder particles continued to decrease in size to 1–3 μm. The particle size became quite uniform, with some particles being larger in size due to adhesion of the powder particles caused by cold welding.

**Figure 5:**
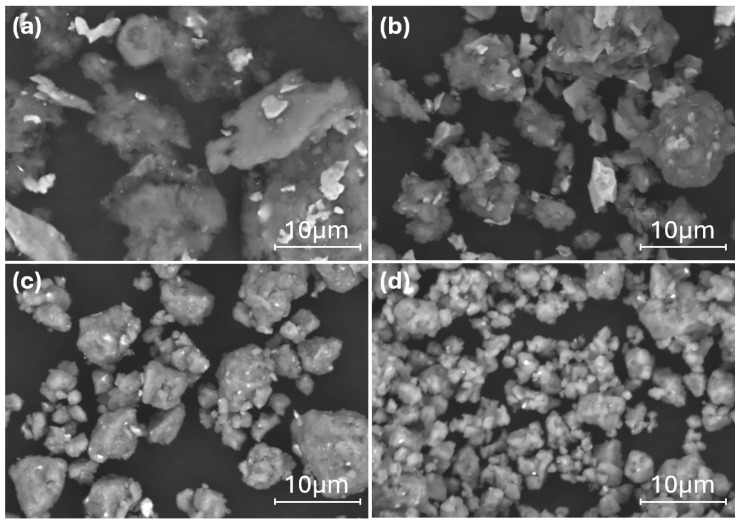
SEM micrographs of the Al_82_Fe_14_Nb_2_Mn_2_ powders milled for (**a**) 1 h, (**b**) 2 h, (**c**) 5 h, and (**d**) 10 h.

Figure 5 presents SEM images of the Al_82_Fe_14_Nb_2_Mn_2_ powder after different milling times. The microstructural evolution of the Al_82_Fe_14_Nb_2_Mn_2_ alloy was similar to that of the Al_82_Fe_16_Nb_2_ alloy. In the early stage of the milling process (1 h of milling), the mixture powders became deformed due to collisions with the milling ball and the vial (Figure 5a). Powders of Al, Fe, Nb, and Mn were laminated after impact. At this stage, the cold-welding process dominated the adhesive metal powders comprising the layer structure. The white and dark phases were adhesive and laminated. The size of the as-milled powders was about 50 μm. With an increase in the milling time to 2 h, the milled powder experienced a fragmentation process, reducing the particle size to approximately 5–15 μm (Figure 5b). Two phases of light and dark could still be observed in the milling powder, indicating that, under a milling time of 2 h, the homogenization process had not yet entirely occurred. The decrease in particle size was evident with a reduction in crystal size due to the diffraction peak being broader than that of the sample after 1 h of milling (Figure 3b). With an increase in the milling time to 5 h, the fragmentation process dominated the powder size by about 10 μm (Figure 5c), with particles showing a more uniform mixture compared to the results under the 2 h milling process. According to the XRD patterns in Figure 3b, further crystal size reductions (due to the diffraction peak being broader than that of the 2 h as-milled sample) coincided with the formation of a new BCC1 phase solid solution. Thus, diffusion occurred when the milling time was 5 h, forming the BCC1 solid solution. By extending the milling time to 10 h (Figure 5d), the powder grains continued to decrease in size to 1–3 μm. Most particles were uniformly sized, although some larger particles appeared due to the adhesion of the powder particles caused by cold welding. The alloy powder was almost completely amorphous, leaving only a few broadened diffraction peaks that are characteristic of nanocrystals, as shown in Figure 3b.

**Figure 6:**
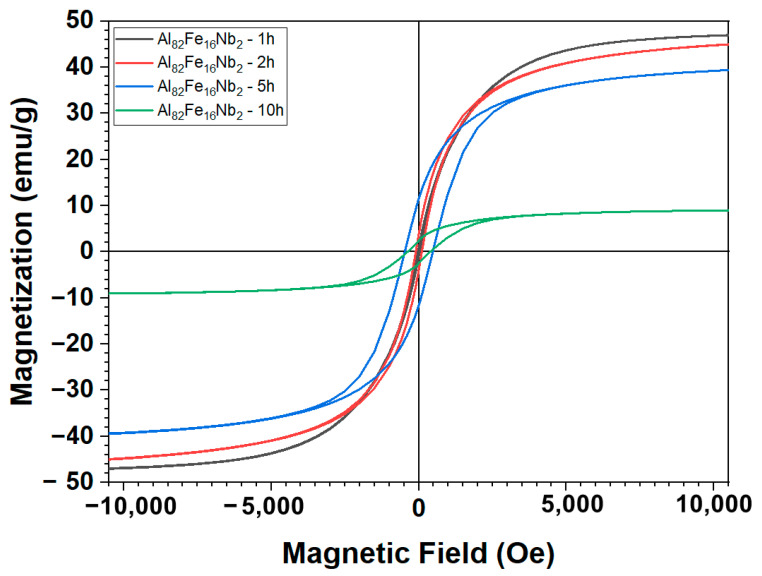
Hysteresis curves of Al_82_Fe_16_Nb_2_ powders milled for different milling times.

**Figure 7:**
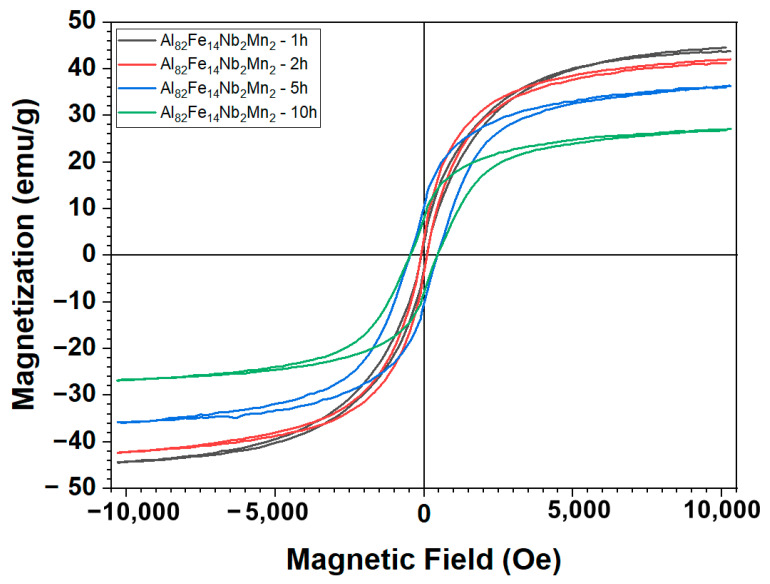
Hysteresis curves of Al_82_Fe_14_Nb_2_Mn_2_ powders milled for different milling times.

Figure 6 and Figure 7, respectively, present the hysteresis magnetic curves of Al_82_Fe_16_Nb_2_ and Al_82_Fe_14_Nb_2_Mn_2_ alloys milled at 350 rpm for 1 h, 2 h, 5 h, and 10 h. Here, all curves are sigmoidal in shape, indicating incomplete saturation at external fields as high as 10,000 Oe. The values of saturated magnetization (M_s_) and magnetic resistance (H_c_) for alloys Al_82_Fe_16_Nb_2_ and Al_82_Fe_14_Nb_2_Mn_2_ with different milling times are listed in Table 3. For the Al_82_Fe_16_Nb_2_ powder, after milling for 1 h, the Ms value was about 47.60 emu/g and decreased to 9.21 emu/g after 10 h of milling. This decrease in the M_s_ values of powders after milling was due to changes in the chemical composition, the local environment of the magnetic atoms, and the electronic structure of the materials, as reported by Xu et al. [29]. As noted by Oanh et al., the formation of an amorphous phase tends to reduce the M_s_ value [22]. Moreover, a reasonably low M_s_ value of about 0.8 emu/g was obtained for the Al_82_Fe_14_Ni_2_Y_2_ alloy. Replacing 2 at.% Mn with Fe in Al_82_Fe_16_Nb_2_ strengthens the electronic interaction of Fe–Mn compared to that of Fe–Fe. Therefore, the M_s_ value of of the final product of the Al_82_Fe_14_Nb_2_Mn_2_ alloy was higher than that of the Al_82_Fe_16_Nb_2_ alloy. In contrast to the decrease in the value of M_s_, with an increase in milling time, the H_c_ values of the Al_82_Fe_16_Nb_2_ and Al_82_Fe_14_Nb_2_Mn_2_ alloy powders increased. By increasing the milling time from 1 to 10 h, the H_c_ of the Al_82_Fe_16_Nb_2_ alloy increased from 23.94 to 383.79 Oe, while the H_c_ of the Al_82_Fe_14_Nb_2_Mn_2_ alloy increased from 86.5 to 456 Oe. The increase in H_c_ during milling is attributable to the use of high-energy milling, which produces a high deviation density and residual stress in the milling powder [30,31,32,33]. For the Al_82_Fe_16_Nb_2_ alloy system, when the milling time is increased from 5 h to 10 h, the magnetic resistance decreases from 474.65 to 383.79 Oe due to a reduction in the particle size and an increase in the proportion of the amorphous phase. However, for the Al_82_Fe_14_Nb_2_Mn_2_ alloy after milling for 5 h to 10 h, the H_c_ value remained unchanged at 456 Oe. Overall, the Al_82_Fe_16_Nb_2_ and Al_82_Fe_14_Nb_2_Mn_2_ alloys have soft magnetic properties at lower milling times (from 1 h to 5 h) and hard magnetic properties when forming amorphous phases (5 h and 10 h milling).

**Figure 8:**
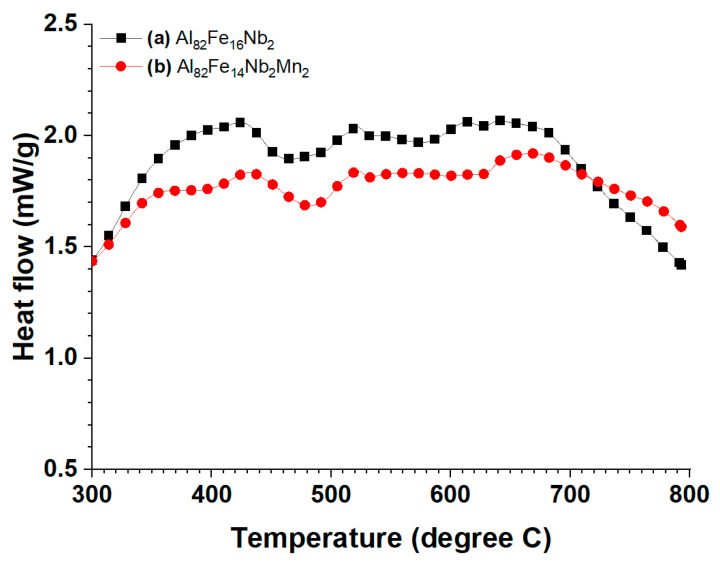
DSC curves of the powder compositions (**a**) Al_82_Fe_16_Nb_2_ and (**b**) Al_82_Fe_14_Nb_2_Mn_2_. Powders were milled for 10 h, and DSC was performed after heating from room temperature to 800 °C.

Figure 8 presents the DSC curves of the Al_82_Fe_16_Nb_2_ and Al_82_Fe_14_Nb_2_Mn_2_ alloys after 10 h of milling. Here, there are exothermic peaks in the temperature range of 300–800 °C. The substitution of 2 at.% Mn for Fe leads to similarities between the two alloys in terms of shape and the onset crystallization temperature (Table 4). As Kuen-Shan Jaw [34] reported, stearic acid decomposes significantly in a temperature range of 320–480 °C.

**Figure 9:**
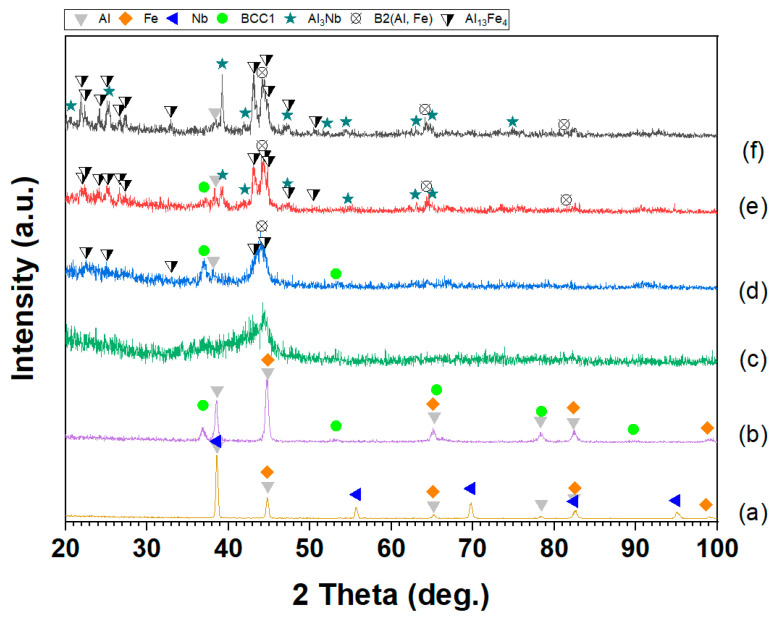
XRD patterns for Al_82_Fe_16_Nb_2_: (**a**) a mixture of powders (Al, Fe, and Nb); powders milled for (**b**) 2 h and (**c**) 10 h, followed by heat treatment for 15 min at (**d**) 550 °C, (**e**) 600 °C, and (**f**) 700 °C.

**Figure 10:**
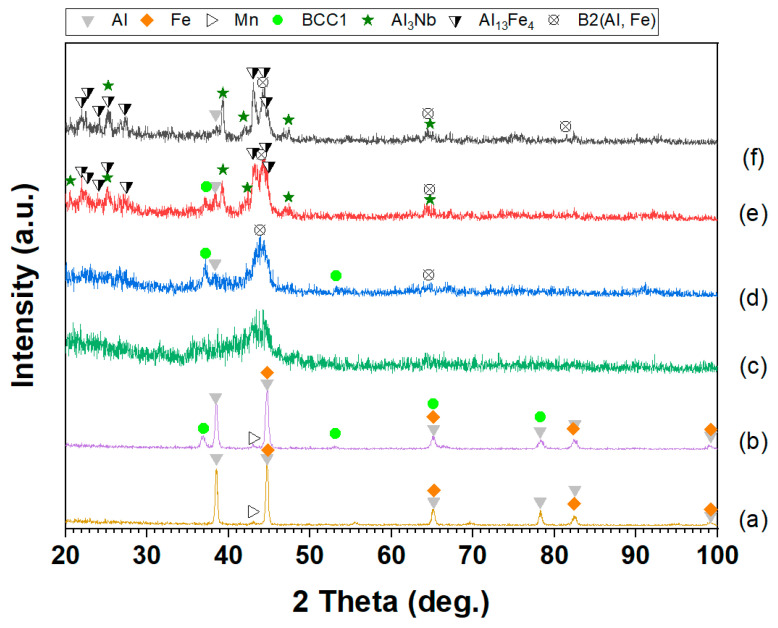
XRD patterns for Al_82_Fe_14_Nb_2_Mn_2_: (**a**) a mixture of powders (Al, Fe, Nb, and Mn); powders milled for (**b**) 2 h and (**c**) 10 h, followed by heat treatment for 15 min at (**d**) 550 °C, (**e**) 600 °C, and (**f**) 700 °C.

To investigate the structural changes in the Al_82_Fe_16_Nb_2_ and Al_82_Fe_14_Nb_2_Mn_2_ alloy powders after 10 h of milling, the alloys were annealed at 550, 600, and 700 °C for 15 min. The annealing temperature was selected based on the onset crystallization temperatures presented in Table 4. The XRD patterns of the Al_82_Fe_16_Nb_2_ and Al_82_Fe_14_Nb_2_Mn_2_ alloy powders after annealing at different temperatures are presented in Figure 9 and Figure 10, respectively. After annealing the Al_82_Fe_16_Nb_2_ and Al_82_Fe_14_Nb_2_Mn_2_ amorphous alloy powder at 550 °C, the diffraction peaks belonged to the Al phase, the solid solution phases of BCC1 and B2 (Al, Fe), Al_13_Fe_4_, and the amorphous phase (Figure 9d and Figure 10d). When the annealing temperature was increased to 600 °C, the phases remained similar to those observed at 550 °C; however, the B2(Al, Fe) phase content and the amorphous phase decreased, and an Al_3_Nb phase appeared (Figure 9e and Figure 10e). At the highest annealing temperature of 700 °C, both the amorphous phase and solid solution B2(Al, Fe) completely decomposed, and the phase content of Al_3_Nb and Al_13_Fe_4_ increased (Figure 9f and Figure 10f).

The ball-milled powders produced in this study, characterized by a relatively refined particle size and uniform distribution, show promising potential for sintering applications, enabling the creation of dense, homogeneous components [35,36]. Fine and evenly dispersed powder particles may promote particle bonding and densification in the sintering process, resulting in improved microstructural properties. Future studies on the sintering of these ball-milled powders could focus on optimizing parameters to maximize the density and homogeneity, thereby broadening their range of applications.

## 4. Conclusions

The full amorphous phase was obtained after 10 h of milling of Al_82_Fe_16_Nb_2_ alloy powders, while a partly amorphous phase was observed after the annealing of Al_82_Fe_14_Nb_2_Mn_2_ alloy powders. The XRD results of the as-milled powders of the alloys appeared as a BCC solid solution phase (BCC1) with a crystalline size of 13–14 nm. Both alloys presented a uniform particle size of about 1–3 μm after milling for 10 h, with some larger particles retained due to agglomeration.

The magnetization curves for the milled and annealed powders were sigmoidal. The values of M_s_ decreased, while H_c_ changed with the milling time in the range of 1–10 h for the milled powder. Al_82_Fe_16_Nb_2_ and Al_82_Fe_14_Nb_2_Mn_2_ alloys have soft magnetic properties at lower milling times (from 1 h to 5 h) and hard magnetic properties when they form amorphous phases (5 h and 10 h of milling).

The DSC curves of the two alloys, Al_82_Fe_16_Nb_2_ and Al_82_Fe_14_Nb_2_Mn_2_, presented exothermic peaks in the temperature range of 300–800 °C. After the Al_82_Fe_16_Nb_2_ and Al_82_Fe_14_Nb_2_Mn_2_ amorphous alloy powders were annealed at 550 °C, the diffraction peaks corresponded to the Al phase, BCC1, B2(Al, Fe), Al_13_Fe_4_, and the amorphous phase. The content of B2(Al, Fe) and the amorphous phases decreased together with the formation of the Al_3_Nb phase after the amorphous powders were annealed at 600 °C. Lastly, the amorphous and B2(Al, Fe) phases completely decomposed at 700 °C.

## Figures and Tables

**Table 2:** Crystallite size of the solid solution phase BCC1.

Alloy System	Milling Time (h)	2θ (°)	Crystal Size (nm)
Al_82_Fe_16_Nb_2_	2	36.8	14.8
Al_82_Fe_16_Nb_2_	5	36.8	13.4
Al_82_Fe_14_Nb_2_Mn_2_	2	36.8	14.8
Al_82_Fe_14_Nb_2_Mn_2_	5	36.7	15.1

**Table 3:** M_s_ and H_c_ values obtained from the VSM analyses for Al_82_Fe_16_Nb_2_ and Al_82_Fe_14_Nb_2_Mn_2_ alloys milled for different times.

Properties	Al_82_Fe_16_Nb_2_			
	1 h	2 h	5 h	10 h
H_c_ (Oe)	23.94	101.59	474.65	383.79
M_s_ (emu/g)	47.60	46.21	40.28	9.21
	Al_82_Fe_14_Nb_2_Mn_2_			
H_c_ (Oe)	86.50	104	456	456
M_s_ (emu/g)	44.50	42.16	36.12	26.93

**Table 4:** Crystallization temperature of Al_82_Fe_16_Nb_2_ and Al_82_Fe_14_Nb_2_Mn_2_ alloys after milling for 10 h.

Sample	T_x1_	T_p1_	T_x2_	T_p2_	T_x3_	T_p3_
Al_82_Fe_16_Nb_2_	496	522	580	613	628	641
Al_82_Fe_14_Nb_2_Mn_2_	494	522	-	-	628	-

## Data Availability

All data supporting the findings of this study are included in the article.
